# Far infrared irradiation suppresses experimental arthritis in rats by down-regulation of genes involved inflammatory response and autoimmunity

**DOI:** 10.1016/j.jare.2021.08.015

**Published:** 2021-09-01

**Authors:** Xi Chen, Hui Zhang, Wu Zeng, Nick Wang, Hang Hong Lo, Chi Kio Ip, Li Jun Yang, W.L. Wendy Hsiao, Wai Man Sin, Chenglai Xia, Betty Yuen Kwan Law, Vincent Kam Wai Wong

**Affiliations:** a Dr. Neher’s Biophysics Laboratory for Innovative Drug Discovery, State Key Laboratory of Quality Research in Chinese Medicine, Macau University of Science and Technology, Macau, SAR China; b Nick Wang Technology Limited, TML Tower, 3 Hoi Shing Road, Tsuen Wan, Kowloon, Hong Kong; c School of Life & Medical Sciences, University College London, London, UK; d Department of Chinese Medicine, Kiang Wu Hospital, Macau, SAR China; e Affiliated Foshan Maternity & Child Healthcare Hospital, Southern Medical University, Foshan 528000, China

**Keywords:** Far infrared irradiation (FIR), Adjuvant-induced arthritis (AIA), Inflammatory response, Autoimmunity, Transcription factors

## Abstract

**Introduction:**

Far-infrared radiation (FIR) is widely used in the treatment of various diseases such as insomnia and cardiovascular risk. Rheumatoid arthritis (RA) is a chronic systemic autoimmune disease in which the therapeutic potential of FIR in RA is unclear.

**Objectives:**

To determine the therapeutic potential and mechanistic actions of FIR in treatment of RA.

**Methods:**

Adjuvant-induced arthritis (AIA) rat models were established to assess the therapeutic potency of FIR in RA treatment. The scoring parameters such as arthritis score, swelling of the hind paw, spleen and thymus indices, micro-CT analysis indices were adopted to estimate the beneficial effects of FIR during RA treatment in AIA model. PCR gene expression arrays were used to analyze inflammatory and autoimmune genes expression profiles in rat synovium. The inflammatory and immunity genes profiling was further analyzed through transcription factor prediction using PROMO. A signaling network map of possible molecular circuits connecting the identified differential genes to the RA's pathogenesis was constructed based on extensive literature reviews, and the major signaling pathways were validated by Western blotting.

**Results:**

Thirty minutes of FIR treatment significantly improved the symptoms of AIA in rats. Gene expression profiling indicated that 27 out of 370 genes were down-regulated by FIR. AP-1, CEBPα, CEBPβ, c-Fos, GR, HNF-3β, USF-1, and USF-2 were predicted as key transcription factors that regulated the identified differential genes. In addition, MAPK, PI3K-Akt, and NF-κB signaling are the major molecular pathways down-regulated by FIR treatment.

**Conclusion:**

FIR may provide beneficial effects on the AIA rat model of arthritis by suppression of the MAPK, PI3K-Akt and NF-κB signaling pathways. Therefore, we believe that FIR may provide an alternative non-pharmacological and non-surgical therapeutic approach for the treatment of RA.

## Highlights

• FIR treatment improved adjuvant arthritis in rats.
• FIR exposure inhibited the inflammatory genes expression of synovial tissues in AIA rats.
• FIR exposure down-regulated inflammatory genes expression mainly through transcription factors AP-1, CEBPα, CEBPβ, c-Fos, GR, HNF-3β, USF-1, and USF-2.
• FIR irradiation may exhibit anti-arthritic effects through inactivation of the MAPK, PI3K-Akt, and NF-κB signaling pathways.

## Graphical abstract

**Figure:**
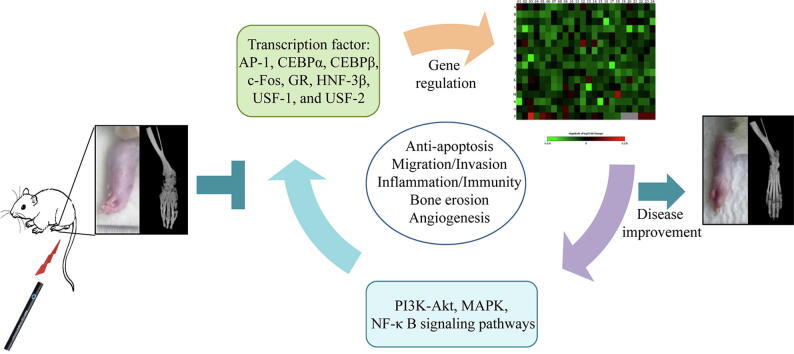

## Introduction

Rheumatoid arthritis (RA) is a chronic systemic autoimmune disease and has become an important public health problem with a global incidence rate of approximately 0.6–1% [1], [2]. RA can occur at any age, with individuals between the ages of 40 to 60 constituting the high-risk group. The incidence rate of females is significantly higher than that of males. The pathophysiology of RA involves chronic synovitis, which may lead to erosive articular cartilage and near-articular bone destruction caused by fibroblast proliferation and immune cell infiltration. These events promote leukocyte recruitment, immune cell activation, the production of inflammatory mediators and proteases, all of which eventually lead to joint deformities and damage [3]. In the early stages of the disease, joints become swollen, tender and warm, and stiffness limits their movement. After a certain period, multiple joints become seriously affected. The small joints of the hands, feet and cervical vertebrae are most commonly involved, but larger joints such as shoulders and knees can also be involved. The long-term disease can affect the heart, lungs, kidneys, other organs and the nervous system. The causes of RA are unclear, though it is generally accepted that a variety of factors, including genetics, environment, gender, and age, are involved in the pathogenesis of RA [4]. It is difficult for patients to sustain long-term medication as the current medication is often associated with severe side effects and resistance. As the disease progressed, high rate of disability, unknown cause, and lack of an effective treatment scheme, patients have to endure the disease for decades, posing a huge threat to human life and health. Disability and the high cost of biological agents also place a heavy financial burden on RA patients and society. Affordable treatment with minimal side effects is essential in the treatment of RA.

Infrared is a kind of electromagnetic radiation with wavelengths longer than visible light. Like all electromagnetic radiation, infrared carries radiant energy. The sunlight that reaches the earth's surface consists of 6% ultraviolet light, 52% visible light and 42% infrared light. This light, and in particular infrared, provide a lot of energy for life on earth [5]. Far-infrared ray (FIR) is a region with wavelengths ranging from 15 μm to 1 mm in the infrared spectrum [6]. FIR can transfer energy between objects by means of thermal radiation. FIR has a strong penetration and radiation force, thus it is easily absorbed and converted into energy by the body [7]. The earth is filled with a narrow range of FIR energy ranging from 5 to 50 µm, and it has been suggested that this narrow range of FIR energy may have contributed to the birth of life. Some studies have shown that FIR can play an important and useful role in animal and cellular models. For example, FIR has a “normalizing effect” on the reproduction, growth, behavior and survival time in mouse model [8]. Similarly, FIR has been shown to improve sleep, blood flow and relieve pain. It has been found that FIR can promote the proliferation of rabbit renal proximal tubular cells *in vitro* by increasing the expression of ATPase Na^+^/K^+^ subunit alpha 1 and glucose transporter 1 and prevent cisplatin-mediated nephrotoxicity [9]. FIR was found to promote tissue healing by enhancing autophagy, inhibit NLRP3 inflammatory microsomal activity in burned skin tissues [10], increase transforming growth factor β1 (TGF-β1) secretion, activate fibroblasts, and promoting collagen regeneration [11], and significantly increase the tensile strength of the skin [12]. Other studies showed that FIR treatment could inhibit RNA levels of IL-6 and TNF-α in lipopolysaccharide-mediated peritonitis in mice [13], and indicate beneficial effect of mice through the regulation of intestinal flora [14]. Despite the numerous health benefits of FIR, the potential effect of FIR on RA is still not known. Therefore, in this study, we aim to evaluate the effect of FIR on adjuvant-induced arthritis in rats, analyze the expression of inflammatory and autoimmune-related genes in rat synovium by RT^2^ Profiler^TM^ PCR Array, and reveal the mechanism behind the effect of FIR on RA.

## Materials and Methods

### Materials

Animals: The Sprague-Dawley rats were purchased from SPF Biotechnology Co.,Ltd (Beijing, China).

Drugs: Methotrexate (MTX) (LC labs, USA) were diluted in the formula (Propylene glycol: Tween 80: Saline = 50:5:45).

Reagents: *Mycobacterium tuberculosis* (#231141, BD, USA); mineral oil (#8042–47-5, Sigma, USA); isoflurane (Aerrane, Baxter Healthcare Corp., USA); TRIzol (#15596026, Ambion, USA); Transcriptor Universal cDNA Master (Roche, USA); PowerUp^TM^ SYBR^TM^ Green Master Mix (#A25742, Applied Biosystems, USA); RT^2^ Profiler^TM^ PCR Array rats Inflammatory Response & Autoimmunity 384HT kit (Qiagen, USA); RIPA (#ab156034, Abcam, UK); Nitrocellulose Transfer Membrane (#66485, Pall Life Sciences, USA); ECL reagents (#1705060, Bio-Rad Laboratories, USA).

Antibodies: Anti-phospho-Akt (1:1000, #4051, Cell Signaling Technology, USA); anti-Akt1/2/3 (1:1000, #sc-81434, Santa Cruz, USA); anti-phospho-Stat3 (1:1000, #sc-8059, Santa Cruz, USA); anti-Stat3 (1:1000, #sc-8019, Santa Cruz, USA); anti-phospho-SAPK/JNK (1:1000, #9255, Cell Signaling Technology, USA); anti-SAPK/JNK (1:1000, #9252, Cell Signaling Technology, USA); anti-phospho-NF-κB p65 (1:1000, #3033, Cell Signaling Technology, USA); anti-NF-κB p65 (1:1000, #8242, Cell Signaling Technology, USA); anti-GAPDH (1:1000, #sc-32233, Santa Cruz, USA); anti-β-actin (1:1000, #sc-47778, Santa Cruz, USA), anti-rabbit HRP-conjugated secondary antibodies (1:4000, #M21002, Abmart, China), and anti-mouse HRP-conjugated secondary antibodies (1:4000, #M21001, Abmart, China).

Apparatus: FIR spectrum emission device (EFFIT LITE®, Hong Kong Nick Wang Technology Limited, China); micro-CT (SkyScan1176, Bruker, Belgium); Nano Drop 2000c spectrophotometer (Thermo Science, USA); ViiA^TM^7 Real Time PCR System (Applied Biosystems, USA); Amersham Imager 600 (GE Healthcare, USA).

Software: NRecon software (Bruker-micro CT, Belgium); ImageJ (NIH, Bethesda, USA); GraphPad Prism 7 software (GraphPad Software, USA).

### Methods

#### FIR spectrum emitting device

In this study, the FIR spectrum transmitter used was EFFIT LITE®. This device can stably emit an FIR spectrum with a wavelength of 4–20 μm and an average photon emissivity of 85.61%.

#### Ethics statement

All experiments involving animals were conducted according to the ethical policies and procedures approved by the Macau University of Science and Technology, Medical Ethics Committee, Macau (China) (Approval no. AL001/DICV/DIS/2019).

#### Establishment and treatment of AIA rat model

The Sprague-Dawley rats (4 per cage) were housed in a constant temperature control room with 12 h light/dark cycles in the Animal Center of the Macau University of Science and Technology and given ad libitum access to food and water. The AIA model was established to inoculate complete Freund's adjuvant (CFA) induced arthritis in male Sprague-Dawley rats, weighing 130 ± 20 g. Non-viable dehydrated *Mycobacterium tuberculosis* (5 mg/mL) was added to mineral oil for emulsification. One hundred microliter of the emulsified oil was injected intradermally into the tail base of the rats, and the first signs of inflammation began to appear gradually on day 9 thereafter. Forty-two male rats were randomly divided into 6 experimental groups as follows: (1) Normal control group (n = 7) without treatments; (2) AIA control group (n = 6), AIA rats were exposed to natural light for 30 min in each experiment; (3) Standard treatment group (n = 5), AIA rats were gavage-fed with MTX 7.6 mg/kg/week in a volume of 10 mL/kg body weight. (4–6) FIR treatment groups. Anesthesia was induced with isoflurane. AIA rats were irradiated with an FIR spectrum emission device for 1 min / day (n = 8), 3 min / day (n = 8), and 30 min / day (n = 8), respectively, on their front and rear paws. The whole experiment ended 27 days after adjuvant injection.

#### Arthritic score

The arthritic score was evaluated and recorded every three days. The severity of paw inflammation was assessed using a qualitative scoring system [15]. Each paw was evaluated and scored individually on a scale from 0 to 4. The scoring criteria was as follows: 0, no swelling or redness in any of the joints; 1, slight swelling and/or redness in small or large joints; 2, moderate swelling and/or redness in one or more small or large joints; 3, severe swelling with redness in large joints and moderate swelling and/or redness in small joints; 4, very severe swelling and redness in large joints and severe swelling and/or redness in the small joints. Additionally, inflammation of the tail was evaluated and scored individually on a scale from 0 to 3. The scoring criteria were as follows: 0, no signs of inflammation, even at the site of injection; 1, injection sites and surrounding tissue exhibit slight edema; 2, ∼25% of the tail is inflammed or exhibits necrotic tissue; 3, >25% of the tail exhibits edema and necrosis. Arthritis score was the sum of paw inflammation score and tail inflammation.

#### Organ indices and Micro-CT analysis

At the end of the 27-days experiment, the rats were euthanized and the organs and hind paws were removed. The thymus and spleen indices were calculated as follows: thymus or spleen index = thymus or spleen weight / body weight × 100. The left hind paws were scanned with micro-CT after 4% PFA fixation. The following scanning parameters were used to obtain high-quality images of the joint of the rat: 35 μm resolutions, 85 kV, 385 μA, 65 ms exposure time, 0.7 min rotation step in 360°, and a 1 mm Al filter. The images were reconstructed using NRecon software. Micro-CT score was obtained from five disease related index of the micro-CT analysis for calcaneus including bone mineral density (BMD), bone volume fraction (BV/TV), cortical mineral density (TMD), trabecular number (Tb.N) and total porosity. Micro-CT score was calculated using the formula as follows: (acquired value - minimum value) / (maximum value - minimum value) or 1- (acquired value - minimum value) / (maximum value - minimum value). The ultimate micro-CT score is equally averaged from the aforementioned five bone-related aspects. The radiological scoring criteria was as follows: 0 = intact bone structure, no bone destruction, clear joint space; 1 = slight bone erosion in any one or two bones; 2 = bone erosion in any three to five bones; 3 = significant bone erosion in more than five bones; 4 = significant bone erosion in almost all metatarsal bones and significant destruction of at least one joint; 5 = no complete bone structure.

#### RNA extraction and cDNA synthesis

Total RNA was extracted from the articular synovium of Sprague-Dawley rats by TRIzol. Determination of RNA concentration by Nano Drop 2000c spectrophotometer. Complementary DNAs were synthesized using Transcriptor Universal cDNA Master.

#### Analysis of inflammatory gene expression profiling

Quantitative PCR was performed using the cDNA prepared with PowerUp^TM^ SYBR^TM^ Green Master Mix and ViiA^TM^7 Real Time PCR System. The RT^2^ Profiler^TM^ PCR Array rats Inflammatory Response & Autoimmunity 384HT kit was assessed according to the manufacturer’s instructions. Results of RT^2^ Profiler^TM^ PCR Array were analyzed by the integrated web-based RT^2^ Profiler^TM^ PCR Array Data Analysis software from Qiagen, which performed calculations based on three independent raw data. The average values of Actb, B2m, Hprt1, Ldha and Rplp1 were used as references, and 2^-△△CT^ method was used to analyze gene expression. All data were analyzed by unmatched one-way ANOVA.

#### Bioinformatics analysis

Detailed information was obtained by searching the NCBI database and the UniProt database for the identified differential genes in the experiment. The promoter regions of the identified differential genes were retrieved from UCSC (http://genome-asia.ucsc.edu/index.html), and the promoter sequences were then entered into PROMO (http://alggen.lsi.upc.es/cgi-bin/promo_v3/promo/promoinit.cgi?dirDB = TF_8.3) to obtain the predicted transcription factors associated with the identified differential genes. Further, pathway analysis of the identified differential genes was performed *via* the KEGG database to investigate their mechanism of action in FIR irradiation therapy against rheumatoid arthritis.

#### Western blotting

The articular synovium of Sprague-Dawley rats was lysed with RIPA. Protein samples were electrophoresed on 8% SDS-PAGE gels and transferred onto nitrocellulose filter membranes. After blocking with 5% non-fat milk and 0.1% Tween 20 in Tris buffered saline solution for 1 h at room temperature, the membranes were incubated overnight at 4 °C with the following primary antibodies. Then, the membranes were incubated with secondary antibodies for 2 h at room temperature. Protein bands were visualized using ECL reagents and Amersham Imager 600, and quantified using the software ImageJ.

### Statistical analysis

Statistical analysis was performed using GraphPad Prism 7 software. The results were expressed as means ± SEM as indicated. The difference was considered statistically significant when the p-value was<0.05. Student’s *t*-test or one-way ANOVA analysis was used for comparison among different groups.

## Results

### Effect of FIR treatment on adjuvant-induced arthritis in rats

**Fig. 1:**
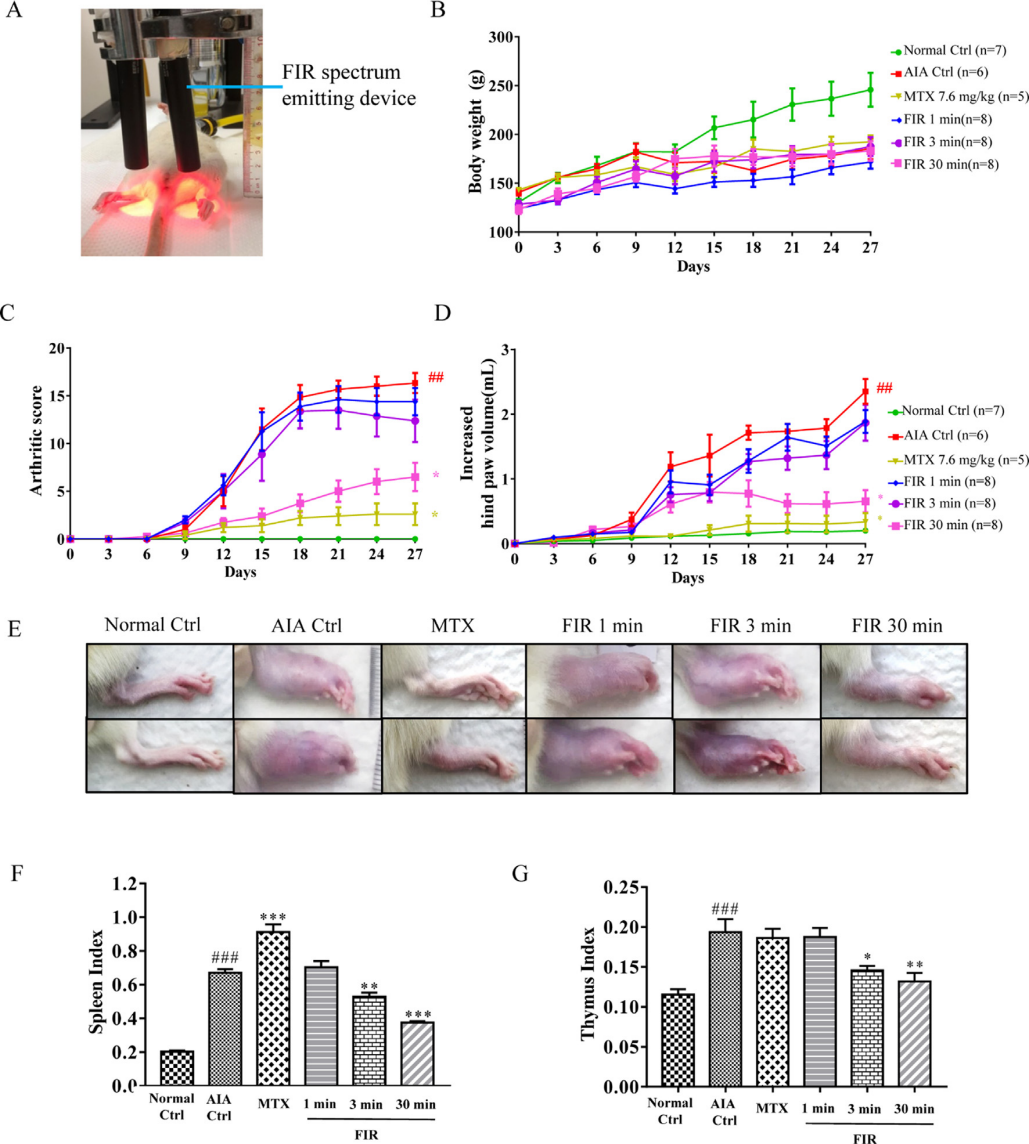
FIR treatment showed anti-arthritis effect on adjuvant-induced arthritis (AIA) in rats. (**A**) Setting of FIR spectrum emitting device. The FIR spectrum emitting device was installed on the bracket and was adjusted to 2 cm above the rat paw. After the rats were anesthetized, their limbs were raised to receive FIR treatment. (**B**) FIR treatment did not affect the body weight of the rats. (**C, D**) FIR decreased the arthritis score and hindpaw swelling of AIA rats. After arthritis induction, rats were split into 6 groups: Normal control group, AIA control group, Standard treatment group (MTX 7.6 mg/kg), 1 min, 3 min, and 30 min FIR-treated groups. Arthritic inflammatory score and hindpaw volume (mL) were measured every 3 days. The whole experiment lasted 27 days. (**E**) Representative images of hindpaw swelling in AIA rats after treatment. (**F, G**) The beneficial effect of FIR on thymus and spleen index of AIA rats. The index of spleen and thymus was expressed as the ratio of wet weight of spleen and thymus to body weight (mg/g). The data are expressed as mean ± SEM (n ≥ 5). * p < 0.05, ** p < 0.01, *** p < 0.001 V.S. the AIA control group. # p < 0.05, ## p < 0.01, ### p < 0.001 V.S. the Normal control group.

Our previous studies demonstrated that FIR modulates gut microbiota and activates G-protein coupled receptors (GPCRs) in mice [14]. Recent studies further indicated that FIR after arthroscopic rotator cuff repair shows promising effects in reducing postoperative pain [16]. However, there is no clear evidence of the impact of FIR treatment on RA; we examine whether FIR treatment can improve the inflammatory conditions of RA. In the current study, AIA rat model was applied to verify the anti-arthritis effect of FIR treatment. As shown in Fig. 1A, the rats were irradiated with FIR for 27 days after the adjuvant induction. The FIR emitting device was fixed on the bracket and adjusted to 2 cm above the rat paw. The AIA rats were then anesthetized, and their limbs were exposed to FIR for 1, 3, and 30 min, respectively. The bodyweight of the irradiated rats showed no significant difference compared to the AIA control group and Standard (MTX) treatment group (Fig. 1B), suggesting that there is no harmful effect on FIR treatment. According to the standard arthritis scoring assessment, we assessed the severity of arthritis starting from day 0. After injection of the adjuvant containing inactivated *Mycobacterium tuberculosis*, the paws of AIA rats rapidly swelled and exhibited distinct clinical syndromes (Fig. 1C-E). In comparison with AIA control group, MTX treatment (7.6 mg/kg/week) significantly inhibited the development of arthritis, as determined by the minimum of arthritic score and hind paw volume (Fig. 1C and D). On the other hand, 1 min and 3 min of FIR treatment demonstrated a milder inhibitory effect on the arthritic symptom. However, AIA rats irradiated with FIR for 30 min showed a significant suppressive effect on the experimental arthritis (Fig. 1C and D). The representative images of the hind paw swelling of AIA rats from each treatment group were shown in Fig. 1E.

Spleen and thymus are crucial organs in the mammalian immune system, and their relative organ weight is an important index for measuring immune function [17]. As shown in Fig. 1F and G, there were significant increases in the spleen and thymus index between the AIA control group and the Normal control group, suggesting the over-activated immune system in experimental arthritis rats. Notably, the AIA rats irradiated with FIR for 3 or 30 min indicated a gradual drop of the spleen and thymus index compared to the AIA control group (Fig. 1F and G), suggesting the recovery of the over-activated immune system after FIR treatment. Nevertheless, MTX treatment in AIA rats further enhanced the spleen index in comparison to the AIA control group (Fig. 1F), indicating the potential adverse effect of MTX treatment.

**Fig. 2:**
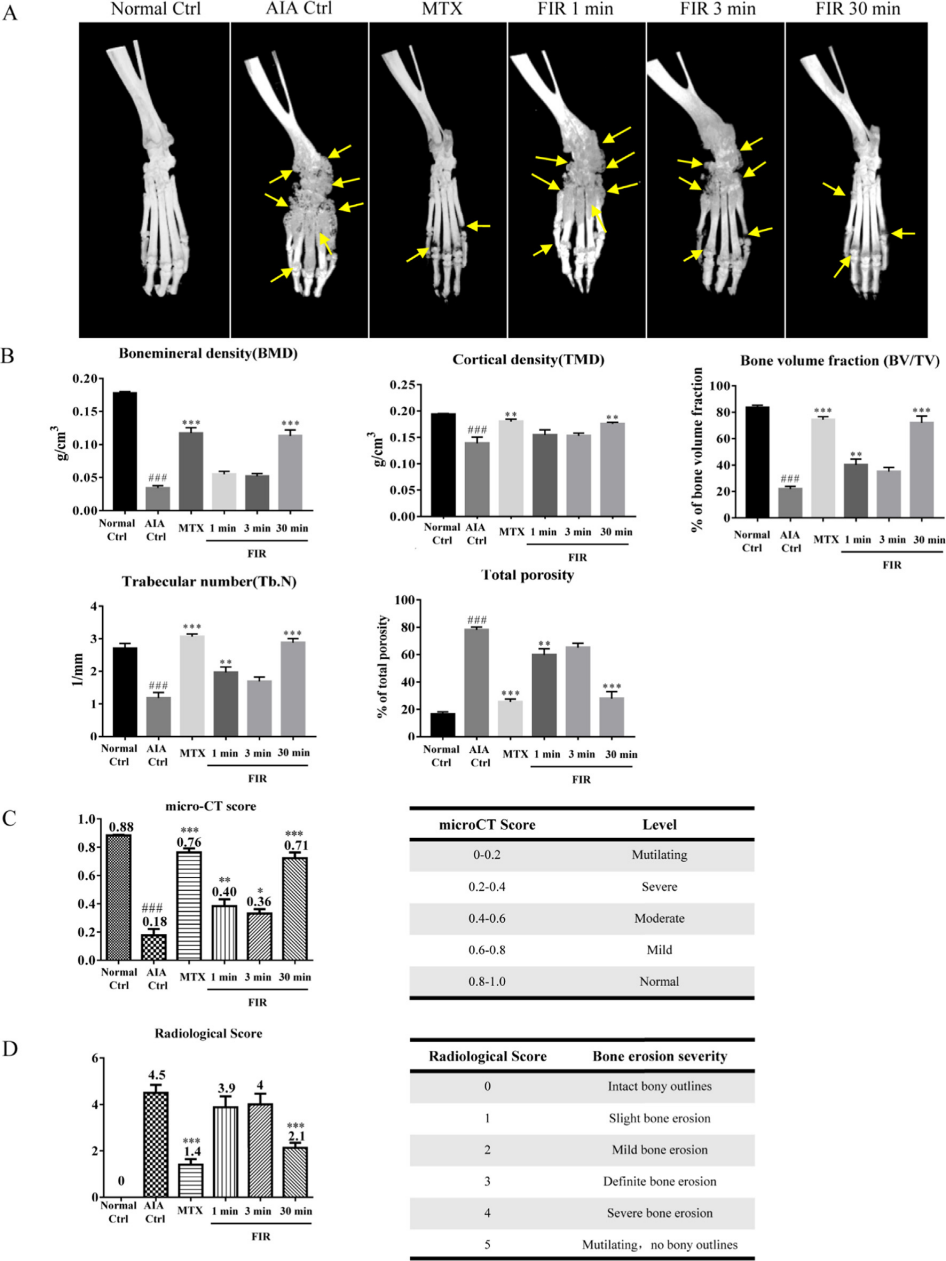
FIR treatment alleviated the destruction of bone and joint tissue in AIA rats. (**A**) Representative micro-CT images of the hindpaw joint of AIA rats after treatment. After 27 days of treatment, the hindpaws of the rats were dissected and micro-CT analysis of bone erosion was performed (indicated by the yellow arrow). (**B**) The bone mineral density (BMD), bone volume fraction (BV/TV), bone cortical mineral density (TMD), trabecular number (Tb.N) and total porosity (total porosity) of the hindpaw joint of AIA rats were measured after treatment. (**C**) Micro-CT score of AIA rats. The micro-CT score was obtained according to five disease-related micro-CT analysis indexes: BMD, BV/TV, TMD, Tb.N and total porosity. (**D**) Radiological score of AIA rats. The Radiological score was obtained according to micro-CT images. The data are expressed as mean ± SEM (n ≥ 5). * p < 0.05, ** p < 0.01, *** p < 0.001 V.S. the AIA control group. # p < 0.05, ## p < 0.01, ### p < 0.001 V.S. the Normal control group.

To determine the protective effect of FIR treatment on articular bone *in vivo*, micro-CT analysis was used to evaluate the bone mineral density, bone volume fraction, cortical mineral density, trabecular number, and the total porosity of the experimental rats. As shown in Fig. 2A, there was a large amount of erosion and destruction of cartilage and bone in the ankle joint of rats in the AIA control group, but no cartilage erosion or bone destruction was found in Normal control rats. Consistent with the trend of hind paw swelling, the destruction of cartilage and bone reduced significantly in the 30 min FIR treatment group (Fig. 2A). Micro-CT results were further analyzed to evaluate the severe swollen joints and bone destruction in the AIA control group rats by direct comparisons of bone mineral density, bone volume fraction, cortical mineral density, trabecular number, and total porosity to other treatment groups. As shown in Fig. 2B and C, the overall micro-CT scores indicated that inflammation and bone destruction were significantly improved with the FIR or MTX treatment. Micro-CT analysis of bone mineral density, cortical density, bone volume fraction, trabecular number, and total porosity indicated that the AIA rats irradiated with 30 min of FIR displayed significantly protection against bone destruction compared to the untreated AIA rats. Of note, the mean micro-CT score of AIA rats with 30 min FIR therapy rose from 0.18 to 0.71, while the mean radiological score dropped significantly from 4.5 to 2.1, representing the inflammatory severity improved from mutilating to mild post FIR treatment (Fig. 2C and D).

### FIR treatment regulates the expression of inflammatory and immunity genes

**Fig. 3:**
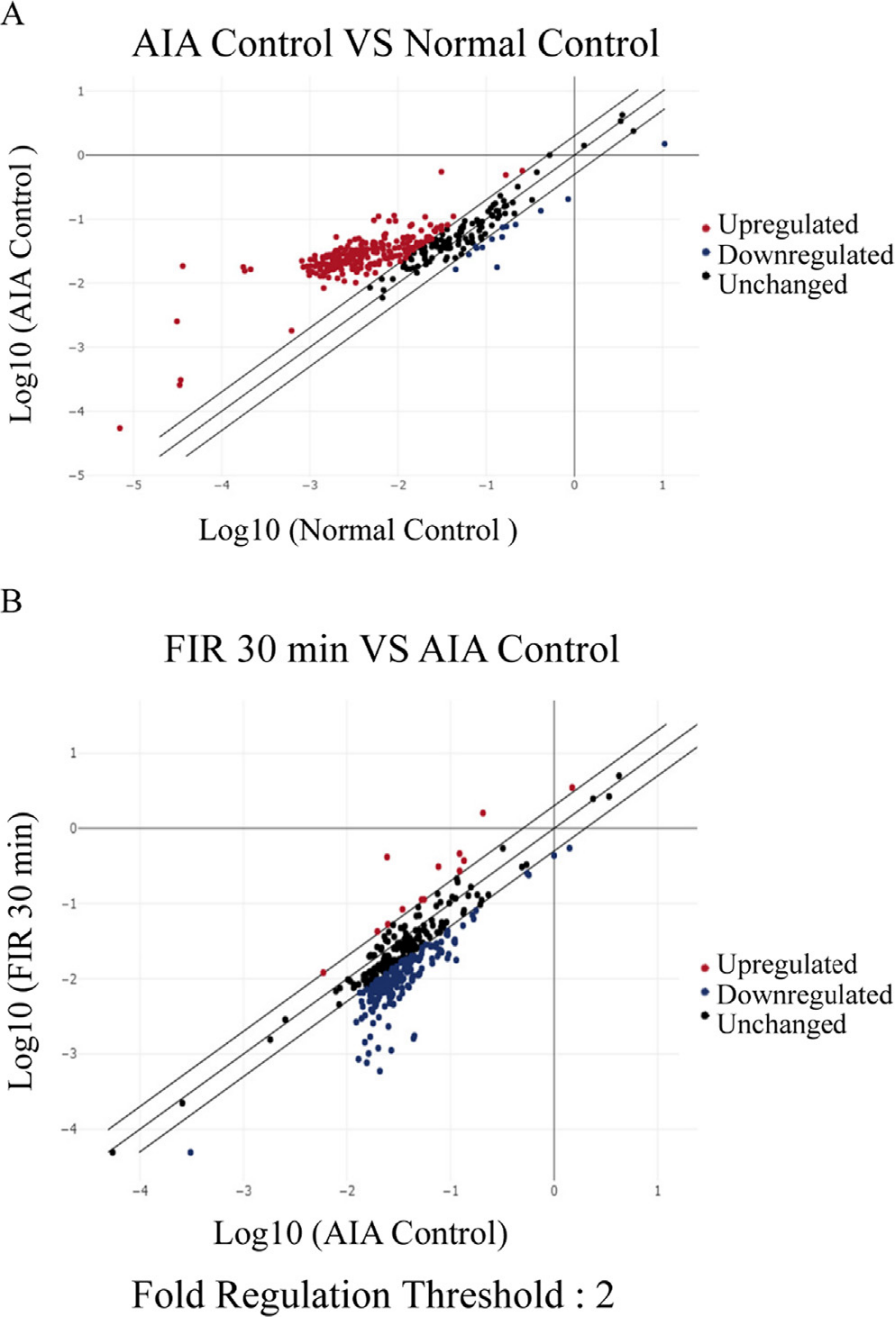
Scatter plot analysis for inflammatory and immunity genes fold regulation from Normal control, AIA control and FIR treatment groups using RT^2^ Profiler PCR Array. (**A**) The synovial tissues of AIA control group and Normal control group were analyzed and compared by RT^2^ Profiler PCR Array. (**B**) The synovial tissues of 30 min FIR treatment group and AIA control group were analyzed and compared by RT^2^ Profiler PCR Array. Based on the results, regulatory scatter maps of inflammatory and immunity genes were obtained. Black spots indicate that the genes are not significantly regulated, red spots indicate the genes were up-regulated, and blue spots indicate the genes were down-regulated. The thresholds of regulated genes are ± 2 fold.

FIR irradiation significantly alleviated arthritic swollen paw and the damage of joint bones. However, the precise FIR-mediated cellular response is unclear. To study the potential anti-inflammatory effect of FIR treatment on arthritis in AIA rats, we utilized RT^2^ Profiler^TM^ PCR inflammatory response and autoimmune gene array to quantitatively analyze the gene expression profile of synovitis in FIR-treated AIA rats. In this study, only genes with ± 2 fold changes of expression in the PCR arrays were included. The results showed that 256 genes were up-regulated, and 14 genes were down-regulated out of the 370 inflammatory and immune genes in the arrays compared to the Normal control group (Fig. 3A). In comparison with the AIA control group, 184 genes were down-regulated, whereas 13 genes were up-regulated in FIR-treated (30 min) AIA rats (Fig. 3B).

**Fig. 4:**
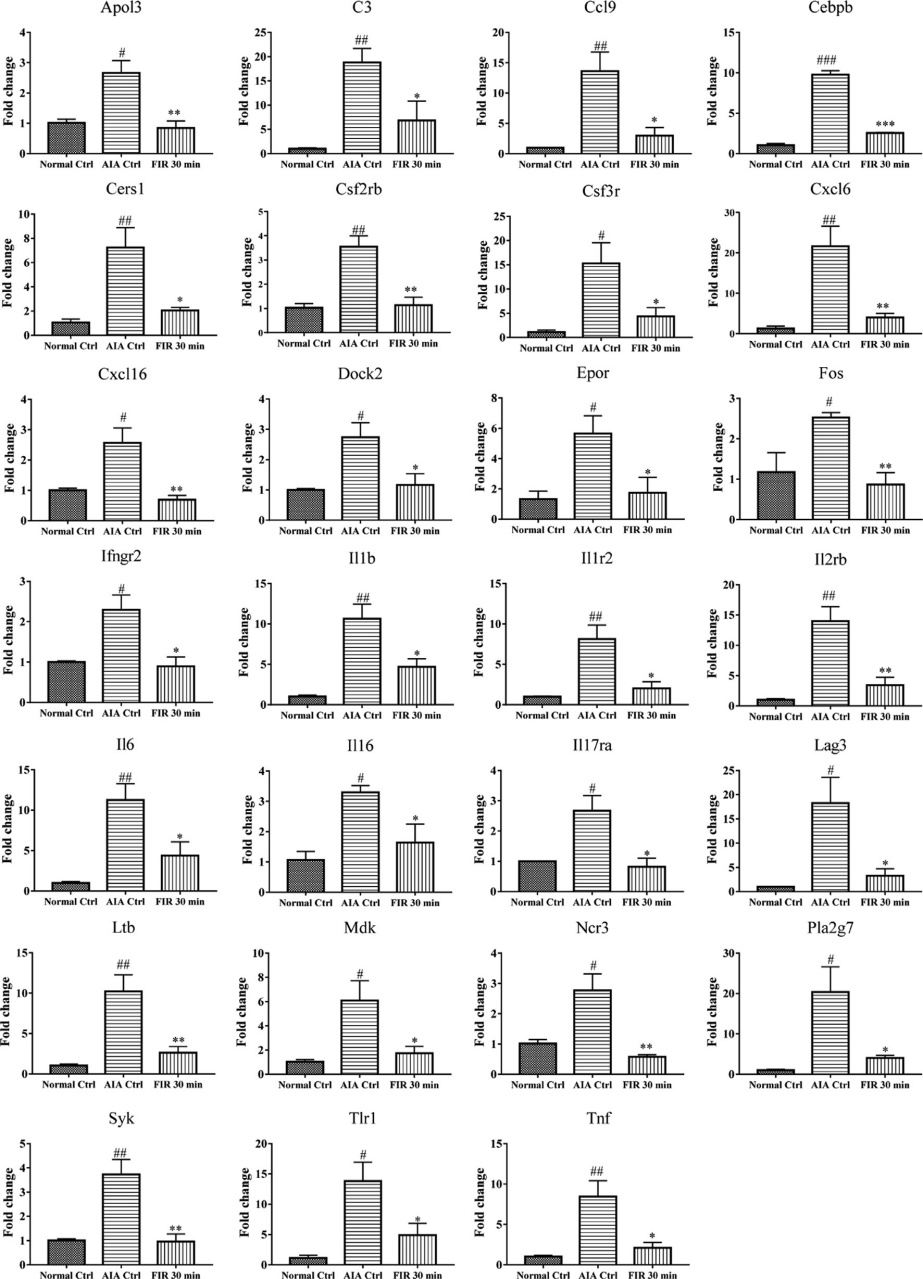
FIR regulated inflammatory and immunity genes expression of articular synovium in AIA rats. RT^2^ Profiler PCR Array was used to analyze the expression of inflammatory and immunity genes in synovium of Normal control group, AIA control group and 30 min FIR-treated group. The average values of Actb, B2m, Hprt1, Ldha and Rplp1 were selected as the reference, and the 2^−ΔΔCT^ method was used to analyze the gene expression. The gene expression was normalized with the AIA control group. The data are expressed as mean ± SEM (n = 3). * p < 0.05, ** p < 0.01, *** p < 0.001 V.S. the AIA control group. # p < 0.05, ## p < 0.01, ### p < 0.001 V.S. the Normal control group.

Further analysis of the three groups (Normal control, AIA control and 30 min FIR-treated groups) of genes which commenced their expression at Ct ≥ 30 were excluded decreasing the number of validated genes to 36. There are 36 inflammatory and immune genes up-regulated in the AIA control group in comparison with the Normal control group (Fig. 4 and Supplementary Fig. S1A). However, the 30 min FIR irradiated-group showed 33 down-regulated genes in comparison with the AIA control group (Fig. 4 and Supplementary Fig. S1B). Accordingly, FIR treatment may inhibit the inflammatory response of AIA rats by down-regulating these overlapped 27 genes (Fig. 4). Meanwhile, the functions of these genes range from upstream cellular receptors, such as Toll-like receptor 1 (TLR1) and interleukin-17 receptor A (IL-17RA), to cytokines such as interleukin-1 β (IL-1β) and interleukin-6 (IL-6), to transcription factors such as CCAAT/enhancer binding protein β (CEBP β), and the roles of these genes in anti-arthritis were classified according to their biological functions (Supplementary Table 1).

### Prediction of transcription factors from inflammatory and immune genes regulated by FIR treatment

**Fig. 5:**
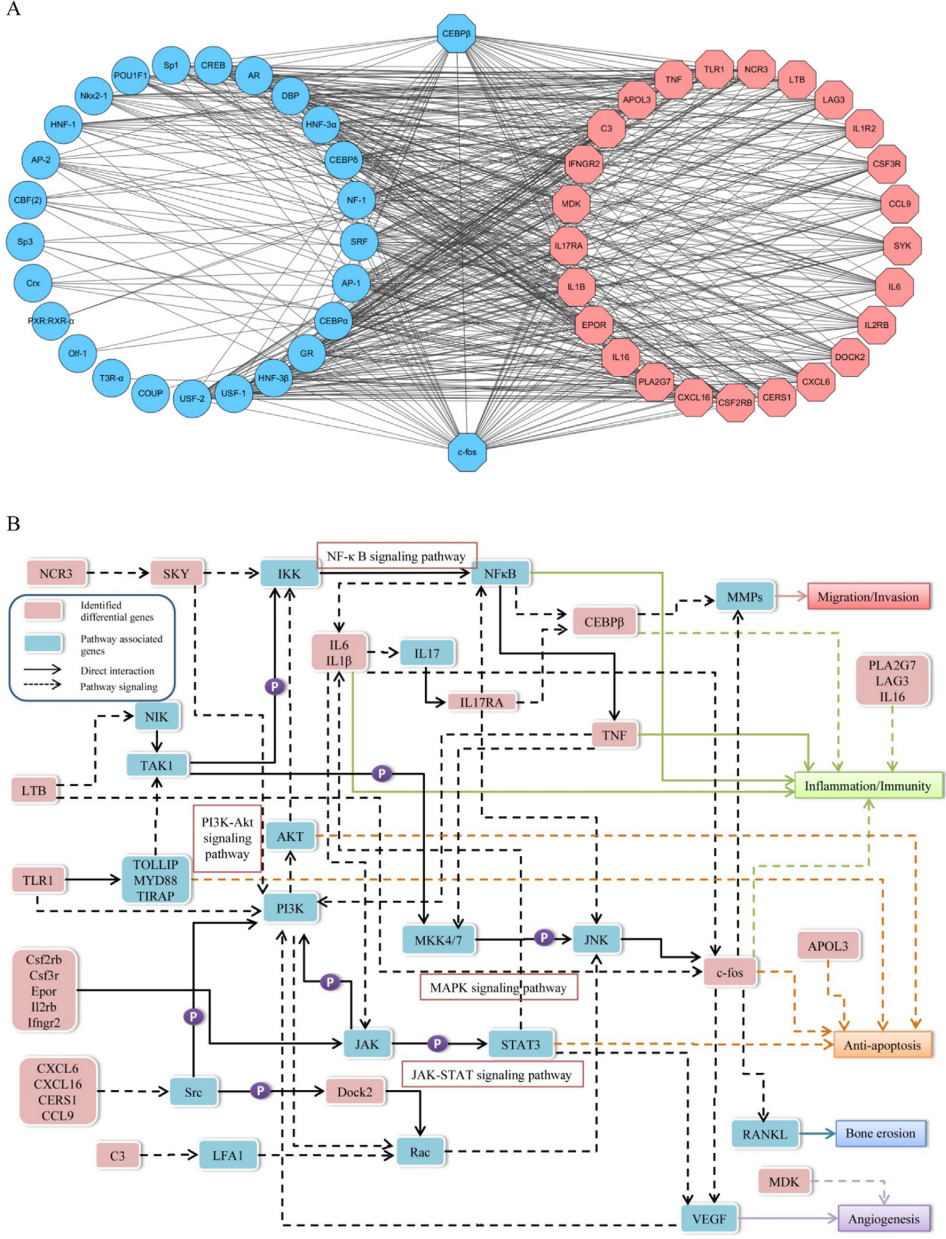
(**A**) The regulatory networks of transcription factors and the identified differential genes were visualized in Cytoscape. Blue nodes represent transcription factors, while octagonal nodes represent the identified differential genes. (**B**) The network map linking the identified differential genes with classical signaling pathways and the pathogenic factors of RA.

Activation of transcription factors such as upstream stimulatory factor 1 (USF-1), CCAAT enhancer binding protein alpha (CEBPα), and POU class 1 homeobox 1 (POU1F1) are crucial determinants of the changes in chromatin landscapes and transcriptomes that specify the consequences of various inflammatory diseases [18]. The identification of transcription factors of inflammatory and immune genes regulated by FIR treatment will provide clues to pursue the anti-arthritic mechanisms of FIR. The promoter regions of the identified genes were acquired using UCSC database, and the transcription factors of promoter regions of these genes were predicted using PROMO. The signal-regulatory networks of the transcription factors -targeted genes are shown in Fig. 5A. Among these transcription factors, AP-1, CEBPα, CEBPβ, c-Fos, GR, HNF-3β, USF-1, and USF-2 were predicted as key transcription factors that regulated the identified differential genes. Of note, CEBPβ and c-Fos were predicted to be the crucial mediators for genes with transcriptional activation and differentiation.

### Construction of a signaling network of FIR-regulated genes associated with RA pathogenesis

To elucidate the functional roles of the identified regulatory genes in response to FIR treatment, extensive literatures review were conducted and these regulated genes were classified according to the key factors etiologically associated with the progression of RA: “immunity and inflammation”, “cell proliferation and apoptosis”, “cell migration and invasion”, “angiogenesis”, and “bone erosion.” By using the KEGG database and the extensive literatures review, it was found that of the 27 differential genes identified, 25 genes regulate inflammation and immune responses, 22 genes are associated with cell proliferation and apoptosis, 22 genes regulate cell migration and invasion, 22 genes promote angiogenesis, and 21 genes facilitate bone erosion. These genes appear to be multifunctional in the pathogenesis of arthritis, and are involved in multiple pathological responses of the arthritic synovium. Accordingly, we created a network diagram containing the identified differential genes (pink boxes) as well as other related genes (blue boxes) and pathways to visually illustrate the relationship of these genes to the development of RA (Fig. 5B).

**Fig. 6:**
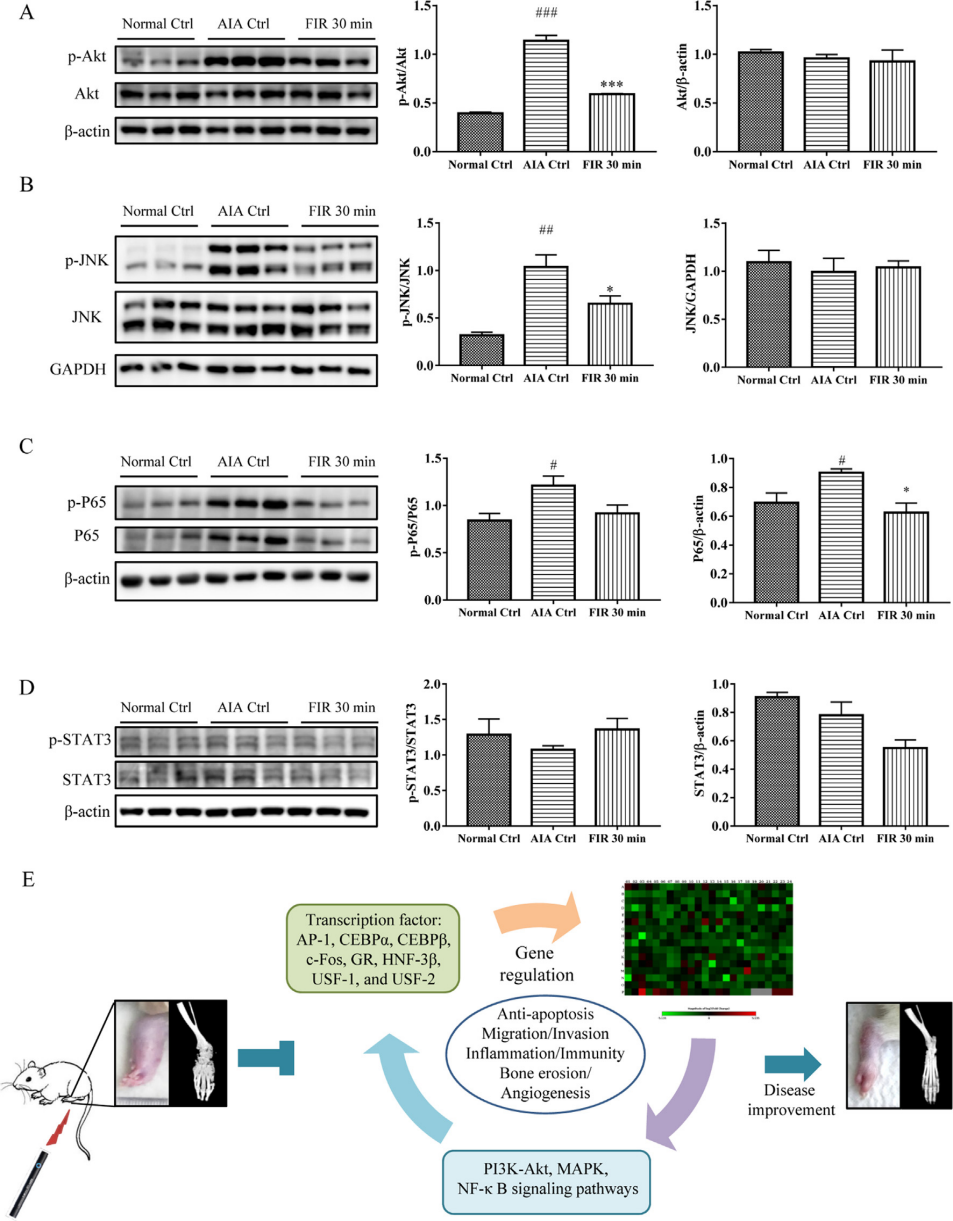
FIR regulated the signaling pathways of Akt, JNK, NF-κB and STAT3 in synovial tissues of AIA rats. (**A**) Western blotting for phosphorylated AKT (p-AKT) and total AKT. (**B**) Western blotting for phosphorylated JNK (p-JNK) and total JNK. (**C**) Western blotting for phosphorylated NF-κB p65 (p-p65) and total p65. (**D**) Western blotting for phosphorylated STAT3 (p-STAT3) and total STAT3. Tissue lysates were prepared from the synovial tissues of Normal control, AIA control group and FIR treatment group. Quantification of phosphorylated form/total form and total form/β-actin or GAPDH were shown after image J analysis. The data are expressed as mean ± SEM (n = 3). * p < 0.05, ** p < 0.01, *** p < 0.001 V.S. the AIA control group. # p < 0.05, ## p < 0.01, ### p < 0.001 V.S. the Normal control group. (**E**) Schematic diagram to show the therapeutic effects and mechanisms of action of FIR in RA treatment.

We finally validated the accuracy of the identified signaling pathways by Western blotting. As shown in Fig. 6A-C, the AIA control group demonstrated a significant increase in the phosphorylated form of AKT, JNK and NF-κB p65 compared with the Normal control group. Of note, the total form of p65 expression was also elevated in the AIA control group. Upon FIR treatment, p-AKT and p-JNK signaling were significantly suppressed (Fig. 6A and B), whereas FIR treatment showed less suppressive effect on p65 activation and mainly inhibited the expression of total p65 (Fig. 6C). Besides, no significant changes in p-STAT3 / total STAT3 and total STAT3 / β-actin were observed (Fig. 6D). Collectively, we concluded that FIR treatment may improve arthritic condition through suppression of the MAPK, PI3K-Akt, and NF-κB signaling pathways.

## Discussion

The application of FIR as a medical treatment with low side effects is growing in various biomedical fields. RA involves persistent autoimmune synovitis and systemic inflammation. Uncontrolled active RA can cause joint injuries, disability, a decline in quality of life, and various serious complications. Notably, FIR can complement ointment in relieving pain caused by arthritis and reducing joint effusion in clinic [19]. Although FIR seems to play some therapeutic role in different animal models of arthritis [20], [21], there is no clear report on the evaluation of the beneficial effects of RA by FIR. Here, we evaluated the beneficial effect of FIR on a classic RA model, the AIA rat model. The results showed that FIR could alleviate the progression of arthritis in AIA rats. Further analysis of synovitis genes expression profile showed that FIR irradiation could markedly inhibit the expression of inflammatory and immunity genes in AIA rats. These results suggest that FIR may have therapeutic potential in RA.

Despite various side effects, MTX is still considered as a first line treatment for RA management. Evidently, 30 min FIR irradiation improved the symptoms of arthritis, similar to that of the Standard (MTX) treatment group. Arthritis scores, swelling of the hind paw, spleen and thymus indices, micro-CT analysis indices and scoring parameters were significantly improved in the FIR-treated AIA rats.

Clinical studies clearly demonstrated that cytokines and chemokines play a pathogenic role in the disease development of RA [22]. Synovitis caused by abnormal immune activation causes joint swelling and injury in RA. The original sparse synovial compartment is infiltrated by immune cells, which release multiple cytokines and chemokines to form a complex immune microenvironment in the synovial compartment. This promotes an abnormal proliferation and an invasive phenotype of synovial fibroblasts [23], [24]. Excessive proliferation of synovial tissue and abnormal immune microenvironment promotes microvascular neovascularization and pannus formation, enhances chondrocyte catabolism, and synovial osteoclast formation promotes joint destruction [25], [26], [27]. Cytokines and chemokines further induce or aggravate the inflammatory response by activating endothelial cells and attracting immune cells to accumulate in the synovial cavity. In addition, adjuvants can also induce different immune cells in the knee joint cavity of arthritis to secrete a large number of cytokines (such as TNF-α, IL-1 β and IL-6) and chemokines (including MCP-1, MIP-1α, and RANTES) [28]. Interestingly, our PCR Array data showed that FIR treatment reduced the expression of cytokines and chemokines in immune cell components collected from the AIA rat model. Furthermore, we determined the mechanism of FIR regulating these inflammatory and immunity genes through transcription factors prediction. Our results predicted that FIR treatment may regulate inflammatory and immunity genes expression through activation of transcription factors such as AP-1, C/EBP α, C/EBP β, c-Fos, GR, HNF-3 β, USF-1, and USF-2.

We have further classified these regulatory genes into the following five categories based on factors associated with RA development: “immunity and inflammation,” “cell proliferation and apoptosis,” “cell migration and invasion,” “angiogenesis,” and “bone erosion.” For instance, the infiltration of the joint by circulating immune cells contributes to the massive release of multiple cytokines and chemokines, leading to synovial inflammation [24], [29]. The abnormal proliferation and impaired apoptosis of arthritic synoviocytes lead to the formation of microvascular neovascularization of the synovial tissue and the formation of pannus, which are important causes of cartilage and bone destruction [16], [30], [31]. Furthermore, systemic multi-joint involvement in RA is associated with abnormal migration and invasive capacity of the arthritic synovium [32]. The molecular pathways mediated by the identified differential genes expression form a complex network in the pathogenesis of RA, and their pathways are connected in series with each other. Accordingly, we have charted a blueprint outlining such potential molecular pathways using signal cascades recorded in kinds of literature to connect those validated genes to the main pathogenesis of RA. The network of regulatory pathways that manipulate cellular functions are mainly through cytokine and cytokine receptor activation of the NF-κB signaling pathway [33], [34], [35], the JAK/STAT signaling pathway [36], [37], [38], the MAPK pathway [39], [40], [41], and the PI3K/AKT signaling pathway [42], [43], [44]. Although such a signaling network is created by converging signaling pathways identified in studies of various cell types, it may potentially be involved in the pathogenesis of RA, which provides key clues for further research into the molecular mechanisms of FIR for the treatment of RA. For instance, Cheng-Shyuan Rau et al. found that FIR could regulate the MAPK signaling pathway through MEK/ERK but not Akt or JNK [45]. However, our study showed that FIR might regulate the MAPK signaling pathway through Akt and JNK in the AIA rat model. Some studies have shown that FIR could up-regulate the level of phosphorylated Akt (p-Akt), reduce apoptosis and cell death, or promote cell proliferation [46], [47], [48]. Hui-Wen Chiu et al. found that FIR could induce autophagy by inhibiting signaling in the Akt pathway and role in skin photoaging in hairless mice [49]. However, we demonstrated that FIR could inhibit the phosphorylation of AKT and slow the progression of arthritis. Besides, Thai-Ha Nguyen Tran et al. showed that exposure to FIR could significantly reduce acute restraint stress in mice by inhibiting JAK2/STAT3 signaling [50]. In contrast, we found that FIR treatment had no significant effect on STAT3 phosphorylation and total STAT3 expression. Furthermore, the NF-κB signaling pathway plays a crucial role in regulating the immune response to infection. Ruiyin Huang et al. found that FIR could inhibit the expression of p65 and reduce endotoxin-induced lung injury and inflammatory reaction in ARDS rats [51]. This study also suggests that the beneficial effect of FIR on arthritis may be through inhibition of p65 expression.

Collectively, it is reasonable to conclude that FIR may provide beneficial effects on the AIA rat model of arthritis by suppression of the MAPK, PI3K-Akt and NF-κB signaling pathways, and down-regulating of inflammatory and immunity genes through key transcription factors such as AP-1, C/EBP α, C/EBP β, c-Fos, GR, HNF-3 β, USF-1, and USF-2 (Fig. 6E). Therefore, we believe that FIR, as a non-pharmacological and non-surgical treatment, provides an alternative approach for RA treatment, and expands the clinical applications to meet patient expectations.

## CRediT authorship contribution statement

**Xi Chen:** Methodology, Validation, Formal analysis, Investigation, Writing - original draft. **Hui Zhang:** Validation, Formal analysis, Investigation. **Wu Zeng:** Validation, Formal analysis, Investigation. **Nick Wang:** Resources. **Hang Hong Lo:** Software, Visualization. **Chi Kio Ip:** Writing - review & editing. **Li Jun Yang:** Writing - review & editing. **W.L. Wendy Hsiao:** Writing - review & editing. **Wai Man Sin:** Writing - review & editing. **Chenglai Xia:** Writing - review & editing. **Betty Yuen Kwan Law:** Conceptualization, Supervision, Project administration, Funding acquisition. **Vincent Kam Wai Wong:** Conceptualization, Supervision, Project administration, Funding acquisition.

## Declaration of Competing Interest

The authors declare that they have no known competing financial interests or personal relationships that could have appeared to influence the work reported in this paper.
