# Supplementary material for: Far infrared irradiation suppresses experimental arthritis in rats by down-regulation of genes involved inflammatory response and autoimmunity
Source: J Adv Res. 2021 Sep 1;38:107–18. doi: 10.1016/j.jare.2021.08.015 (PMC9091720; doi:10.1016/j.jare.2021.08.015)
Supplement: Supplementary data 1 [file mmc1.pdf]

Supplementary Table.1

No.	Gene Symbol	Description	AIA Ctrl VS Normal Ctrl		30 min FIR VS AIA Ctrl	
			Fold Regulation	p-Value	Fold Regulation	p-Value
		Cytokines				
		Chemokines				
1	Ccl9	Chemokine (C-C motif) ligand 9	12.94	0.0075	-6.15	0.0162
		Interleukins				
1	Cers1	LAG1 homolog, ceramide synthase 1	6.80	0.0072	-3.37	0.0158
2	Il16	Interleukin 16	3.28	0.0145	-2.50	0.049
3	Il1b	Interleukin 1 beta	10.34	0.0021	-2.31	0.0205
4	Il6	Interleukin 6	10.93	0.0055	-2.93	0.0321
		Other Cytokines				
1	Ltb	Lymphotoxin beta (TNF superfamily, member 3)	9.83	0.0037	-4.12	0.0093
2	Mdk	Midkine	5.71	0.0205	-3.66	0.0379
3	Tnf	Tumor necrosis factor (TNF superfamily, member 2)	8.04	0.0084	-4.55	0.0169
		Cytokine Receptors				
		Chemokine Receptors				
1	Cxcl16	Chemokine (C-X-C motif) ligand 16	2.45	0.0181	-3.75	0.0078
2	Cxcl6	Chemokine (C-X-C motif) ligand 5	20.35	0.0050	-5.39	0.0100
		Interleukin Receptors				
1	Il17ra	Interleukin 17 receptor A	2.56	0.0227	-3.59	0.0145
2	Il1r2	Interleukin 1 receptor, type II	7.74	0.0071	-4.47	0.0146
3	Il2rb	Interleukin 2 receptor, beta	13.63	0.0020	-4.70	0.0057
		Other Cytokine Receptors				
1	Csf2rb	Colony stimulating factor 2 receptor, beta, low-affinity (granulocyte-macrophage)	3.49	0.0034	-3.47	0.0042
2	Csf3r	Colony stimulating factor 3 receptor (granulocyte)	13.82	0.0167	-4.40	0.0476
3	Epor	Erythropoietin receptor	5.44	0.0317	-4.41	0.0464
4	Ifngr2	Interferon gamma receptor 2	2.23	0.0188	-2.64	0.0134
		Cytokine Metabolism				
1	Cebpb	CCAAT/enhancer binding protein (C/EBP), beta	9.75	<0.0001	-3.80	<0.0001
2	Il1b	Interleukin 1 beta	10.34	0.0021	-2.31	0.0205
3	Il6	Interleukin 6	10.93	0.0055	-2.93	0.0321
4	Syk	Spleen tyrosine kinase	3.63	0.0056	-4.16	0.0051
5	Tlr1	Toll-like receptor 1	13.22	0.0056	-3.15	0.0051
		Cytokine Production				
1	Cebpb	CCAAT/enhancer binding protein (C/EBP), beta	9.75	<0.0001	-3.80	<0.0001
2	Il1b	Interleukin 1 beta	10.34	0.0021	-2.31	0.0205
3	Il6	Interleukin 6	10.93	0.0055	-2.93	0.0321

4	Lag3	Lymphocyte-activation gene 3	16.57	0.0148	-6.01	0.027
5	Syk	Spleen tyrosine kinase	3.63	0.0056	-4.16	0.0051
6	Tlr1	Toll-like receptor 1	13.22	0.0056	-3.15	0.0051
Acute-Phase Response						
1	Cebpb	CCAAT/enhancer binding protein (C/EBP), beta	9.75	＜0.0001	-3.80	＜0.0001
2	Il6	Interleukin 6	10.93	0.0055	-2.93	0.0321
Inflammatory Response						
1	Apol3	Apolipoprotein L, 3	2.60	0.0106	-3.34	0.0066
2	C3	Complement component 3	18.36	0.0078	-3.68	0.0428
3	Dock2	Dedicator of cytokinesis 2	2.66	0.0231	-2.49	0.0348
4	Fos	FBJ osteosarcoma oncogene	2.52	0.0497	-3.24	0.0218
5	Il16	Interleukin 16	3.28	0.0145	-2.50	0.049
6	Il1b	Interleukin 1 beta	10.34	0.0021	-2.31	0.0205
7	Ncr3	Natural cytotoxicity triggering receptor 3	2.64	0.0157	-4.68	0.0055
8	Pla2g7	Phospholipase A2, group VII (platelet-activating factor acetylhydrolase, plasma)	17.93	0.0156	-4.52	0.0318
9	Syk	Spleen tyrosine kinase	3.63	0.0056	-4.16	0.0051
10	Tlr1	Toll-like receptor 1	13.22	0.0056	-3.15	0.0051
11	Tnf	Tumor necrosis factor (TNF superfamily, member 2)	8.04	0.0084	-4.55	0.0169
Humoral Immune Response						
1	C3	Complement component 3	18.36	0.0078	-3.68	0.0428
2	Csf2rb	Colony stimulating factor 2 receptor, beta, low-affinity (granulocyte-macrophage)	3.49	0.0034	-3.47	0.0042
3	Dock2	Dedicator of cytokinesis 2	2.66	0.0231	-2.49	0.0348
4	Il1b	Interleukin 1 beta	10.34	0.0021	-2.31	0.0205
5	Tnf	Tumor necrosis factor (TNF superfamily, member 2)	8.04	0.0084	-4.55	0.0169

Supplementary Table.1. NCBI database was used to classify the inflammatory and immunity genes that have been identified. The gene expression multiple changes of the AIA control group compared with the Normal control group, and 30 min FIR-treated group compared with the AIA control group.

Supplementary Figure S1

A

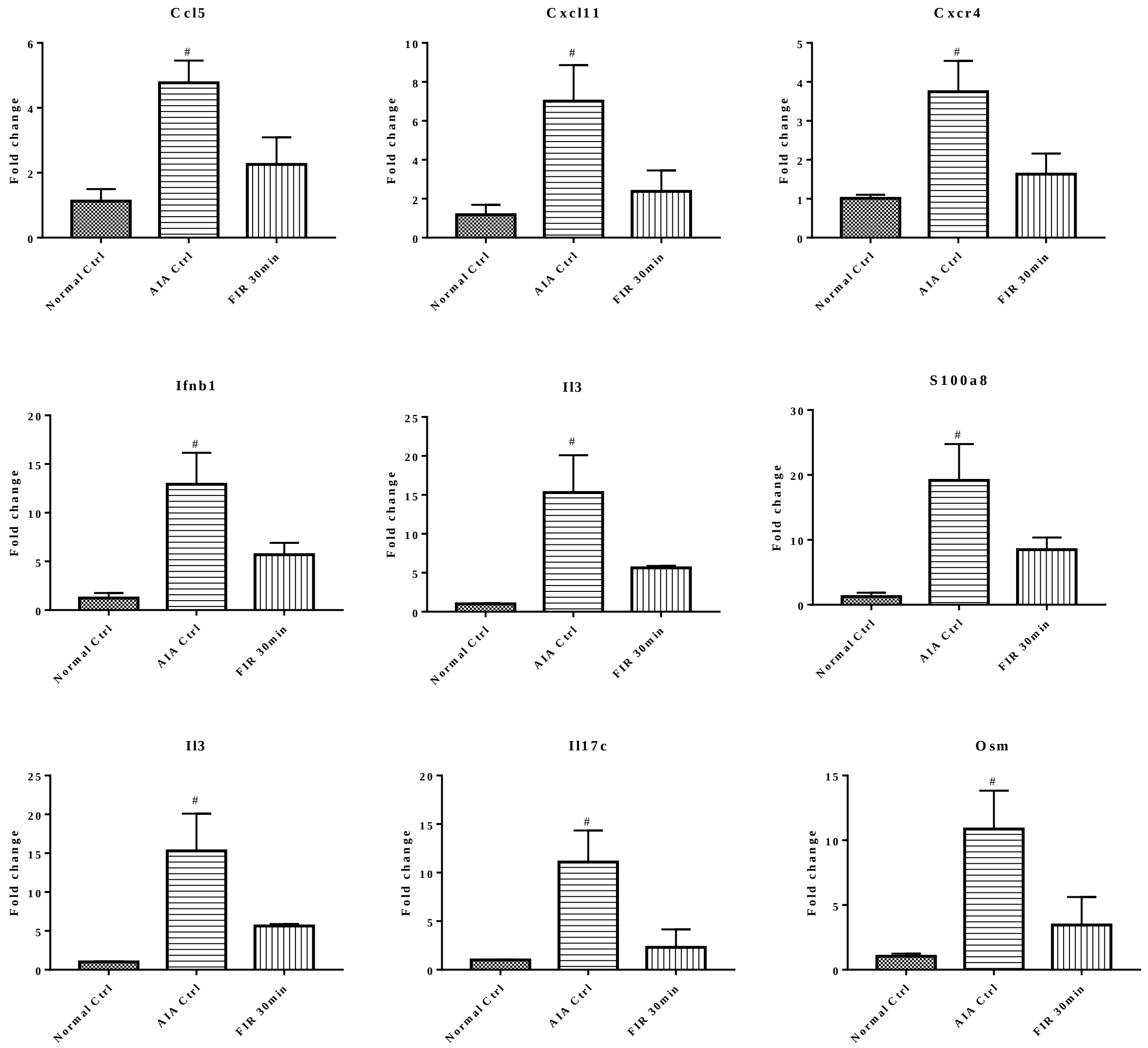

B

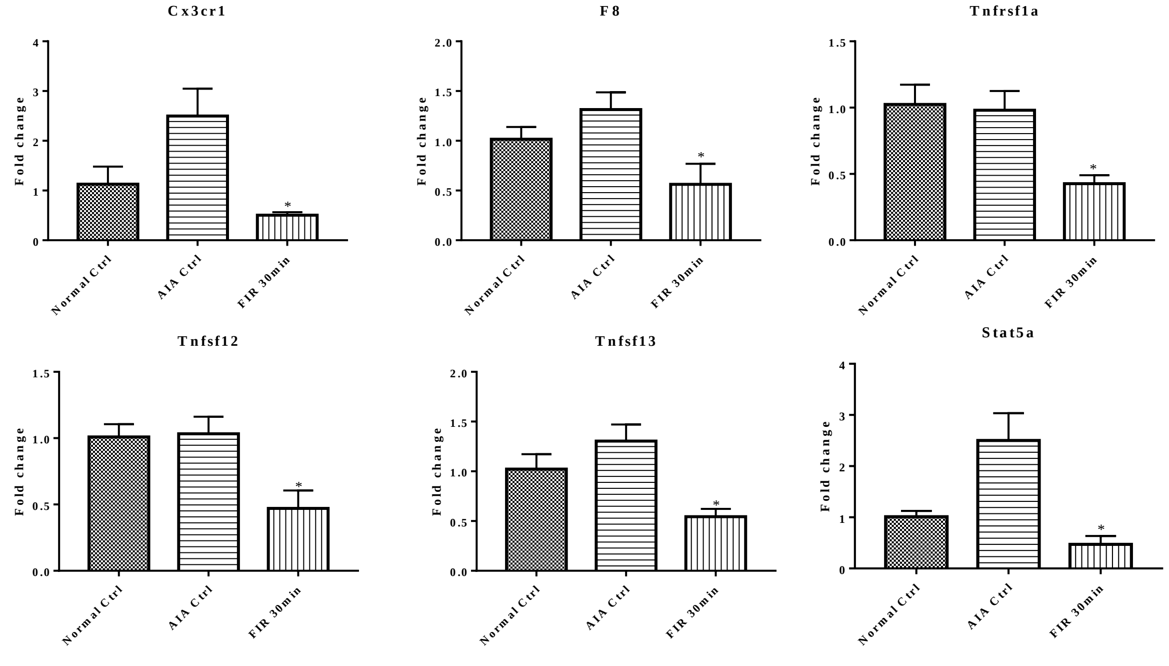

Supplementary Fig. S1. Other inflammatory and immunity genes expression were regulated in response to FIR treatment in AIA rats. (**A**) The expression of genes significantly up-regulated between AIA control and Normal control groups were not significantly suppressed by FIR treatment. (**B**) The expression of genes significantly down-regulated by FIR did not show a significant difference between AIA control and Normal control groups. RT ^{2} Profiler PCR Array was used to analyze the expression of inflammatory and immune genes in synovium of Normal control, AIA control and 30 min FIR-treated group. The average values of Actb, B2m, Hprt1, Ldha and Rplp1 were selected as the reference, and the $2^{-\Delta\Delta CT}$ metho d was used to analyze the gene expression. The gene expression was normalized with the Normal control group. The data are expressed as mean ±SEM (n=3). * $p < 0.05$, ** $p < 0.01$, *** $p < 0.001$, the difference was significant compared with the AIA control group. # $p < 0.05$, ## $p < 0.01$, ### $p < 0.001$, the difference was statistically significant compared with the Normal control group.
